# The mediating effect of depressive syndrome on the relationship between adverse childhood experiences and chronic kidney diseases among middle-aged and older adults

**DOI:** 10.3389/fpubh.2025.1536847

**Published:** 2025-04-03

**Authors:** Xinyao Luo, Ke Wang, Junzhe Ran, Zhuyun Zhang, Yupei Li, Baihai Su

**Affiliations:** ^1^Division of Nephrology, Department of Medicine, West China Hospital, Sichuan University, Chengdu, China; ^2^West China School of Medicine, Sichuan University, Chengdu, China; ^3^The Psychiatric Laboratory and Mental Health Center, West China Hospital, Sichuan University, Chengdu, China

**Keywords:** adverse childhood experiences, chronic kidney disease, depression syndrome, logistic regression, mediation analysis

## Abstract

**Background:**

Chronic kidney disease (CKD) is projected to rank among the top five causes of mortality by 2050. In addition to established risk factors, adverse childhood experiences (ACEs) have recently emerged as significant contributors to health risks, including CKD and depressive syndrome (DS). However, the mechanisms linking ACEs, DS, and CKD remain unclear. This study aims to explore the role of ACEs in CKD development, with a focus on the mediating effects of DS.

**Methods:**

This retrospective cohort study analyzed data from 10,247 participants in the China Health and Retirement Longitudinal Study (CHARLS). Logistic regression models were applied to assess the associations between ACEs, DS, and incident CKD, adjusting for demographic and lifestyle factors. Mediation analysis was conducted to evaluate the role of DS in the relationship between ACEs and CKD.

**Results:**

Logistic regression analysis indicated that participants with a history of ACEs were at higher risk for both DS and CKD. Mediation analysis demonstrated that DS partially mediated the associations between CKD and seven specific ACEs: physical abuse, household substance abuse, household mental illness, domestic violence, unsafe neighborhood, peer bullying, and parental disability. Notably, DS fully mediated the relationship between CKD and unsafe neighborhood.

**Conclusion:**

ACEs significantly influence CKD risk in middle-aged and older adults, with DS serving as a key mediator. These findings underscore the importance of early mental health interventions and ACE-focused preventive strategies to reduce the burden of CKD.

## 1 Introduction

Chronic kidney disease (CKD) is a major contributor to the global disease burden, affecting around 10% of adults worldwide and responsible for an estimated 1.57 million deaths in 2022 (1, 2). Its prevalence is anticipated to place it among the top five causes of mortality by 2050 (1). CKD progression is associated with a wide range of adverse health outcomes, including cardiovascular complications, reduced quality of life, and premature mortality (3, 4). Given its high morbidity and mortality, identifying and mitigating its underlying risk factors is crucial to improving prevention and management strategies.

CKD pathogenesis is understood to be multifactorial, including inflammatory disorder unhealthy lifestyle behaviors, and genetic predispositions (4–6). In addition to traditional risk factors, psychological factors have been linked to CKD development (7, 8). In recent years, however, adverse childhood experiences (ACEs) have emerged as significant contributors to health risk. ACEs denote various stressful experiences that emerge in childhood, involving exposure to abuse (emotional, physical, sexual), neglect (emotional, physical), and household challenges including family substance misuse, mental disorders, criminal activity, intimate partner violence, and parental separation or divorce (9). Experiencing adverse events during childhood has been associated with heightened susceptibility to numerous health conditions in later life, such as cognitive impairment, increased morbidity, physical disabilities, chronic respiratory and gastrointestinal disorders, cardiovascular ailments, and cancer (10–15).

Additionally, ACEs are correlated with several adult mental health disorders, such as anxiety, depression, substance use disorders and attention-deficit/hyperactivity disorders (16–19). These mental health disorders, particularly depression syndrome (DS), are linked to an elevated risk of CKD, indicating a complex interplay between psychological well-being and CKD development (18). The mechanisms connecting ACEs, DS, and CKD are not yet fully understood. A potential pathway involves ACEs triggering chronic psychological stress, which elevates the likelihood of mental health disorders and may subsequently disrupt immune regulation, contributing to CKD vulnerability (10, 20).

In this context, we hypothesize that DS may mediate the relationship between ACEs and CKD, serving as a psychological pathway linking early-life trauma to later-life physical health outcomes. This study aims to examine the role of ACEs in CKD development, focusing on the mediating effects of DS. Understanding these complex interrelations is essential for advancing public health strategies to reduce CKD burden by addressing both psychological and physiological risk factors.

## 2 Methods

### 2.1 Participants and study design

The China Health and Retirement Longitudinal Study (CHARLS) provided data for this study, with its comprehensive study design and sampling methodology previously detailed by Zhao et al. (21). In brief, CHARLS employed a multistage probability sampling approach, recruiting 17,708 participants from 450 villages or communities in 28 Chinese provinces during its baseline phase. Follow-up surveys were conducted biennially, including 2013, 2015, and 2018, with new participants added at each stage. Additionally, the 2014 life history survey collected childhood experience data from participants of the 2011 and 2013 surveys.

**Figure 1:**
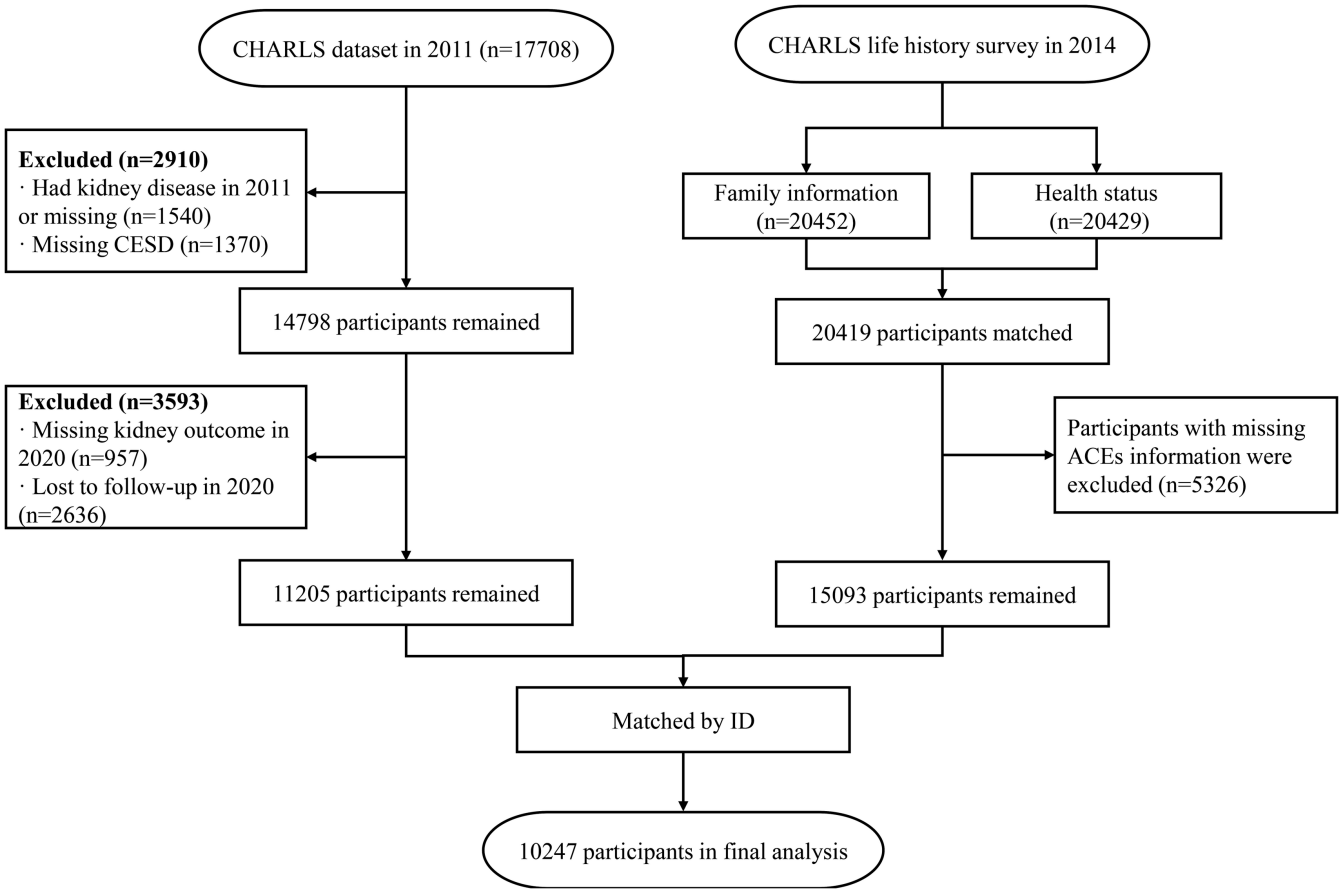
Flow chart of this study. CHARLS, China Health and Retirement Longitudinal Study; CESD, Center for Epidemiologic Studies Depression Scale; ACEs, Adverse childhood experiences.

This research utilized data from the 2011 and 2020 surveys combined with the 2014 life history survey to assess linkages between adverse childhood experiences (ACEs), depressive syndrome (DS), chronic kidney diseases (CKDs), and their comorbid association (DS-CKD). To explore DS’s mediating role between ACEs and CKDs, DS data from 2011 and CKDs data from 2020 were also integrated. We used data from 2011 as baseline surveys. We excluded individuals with pre-existing CKD diagnoses prior to 2011 to ensure that all CKD cases identified in 2020 were newly incident cases. Data analysis was conducted from October 1 to 28, 2023. The workflow of the study is illustrated in Figure 1.

Ethical approval for CHARLS was granted by Peking University’s Institutional Review Board (IRB0000105211015), with all participants providing informed consent. As this study is a secondary analysis of existing datasets, no further ethical clearance or participant consent was required, consistent with established research guidelines. This study followed the Strengthening the Reporting of Observational Studies in Epidemiology (STROBE) guidelines.

### 2.2 Assessment of CKDs

In this study, each patient’s CKD status is represented as a binary variable based on a questionnaire item asking if they had ever been diagnosed with CKD by a doctor. A response of “0” indicates that the respondent did not report a physician-diagnosed condition, while a “1” signifies that the respondent confirmed having received such a diagnosis.

### 2.3 Assessment of ACEs

From the CHARLS dataset, we derived identified ACEs, including 7 standard ACEs (physical abuse, emotional neglect, household substance abuse, household mental illness, domestic violence, household member incarceration, and parental separation or divorce), 2 ACEs from an expanded category (unsafe neighborhood and peer bullying), and 3 additional ACEs cited in prior studies (parental death, sibling death, and parental disability) (10, 12). Details on each ACE indicator and corresponding questionnaire items are available in Supplementary Table S1. Regarding parental and sibling death, the CHARLS life history survey lacked direct questions about parental death during childhood. Consequently, the respondent’s age at parental or sibling death was derived from their birth year and the parent/sibling’s death year. Deaths occurring before the respondent turned 18 were coded as “1.” Responses were dichotomized and aggregated to calculate an individual ACE score for each participant, with total scores ranging from 0 to 12. According to prior epidemiological research (22, 23) and the cumulative numbers of the ACE indicators, participants were categorized into three groups according to their scores: 0 (no exposure), scores below 4 (moderate exposure), and scores of 4 or higher (severe exposure).

### 2.4 Assessment of DS

The Center for Epidemiologic Studies Depression Scale (CESD), originally developed by Radloff in 1977, is a self-report tool designed to assess current DS, especially within primary care settings (24). Due to the length of the original 20-item CESD scale, the CESD-10—a shorter, 10-item version—was later introduced to enable quicker screening (24). The CESD-10 has been widely utilized in older adult populations in China and has been shown to exhibit adequate reliability and validity (25, 26). In CHARLS, participants completed the CESD-10 by self-report. This scale provides a total score ranging from 0 to 30, with scores of 10 or above indicating the presence of DS. In this study, participants with a CESD-10 score above 10 were classified as experiencing DS (1 = presence of DS, 0 = absence of DS) (27).

### 2.5 Other measures

Based on previous research (12) and existing knowledge, in this study, selected demographic and lifestyle data of the participants were collected as covariates, including age (years), gender (female/male), marital status (married/unmarried), education level (below primary school, primary school, middle school, high school or above), smoking history (yes/no), drinking history (yes/no), sleep duration (hours), and life satisfaction scores. The life satisfaction score is a self-assessment of how satisfied a person is with his or her life (Not at all satisfied = 1; Not very satisfied = 2; Somewhat satisfied = 3; Very satisfied = 4; Completely satisfied = 5). In addition, data for these covariates were obtained from the 2011 CHARLS baseline survey.

### 2.6 Statistical analysis

Continuous variables were expressed as means with standard deviations, and categorical variables as frequencies with percentages. Group comparisons employed t-tests or Wilcoxon tests for continuous measures and Chi-square or Fisher’s exact tests for categorical measures. Logistic regression models evaluated associations of ACEs with DS and CKD, adjusted for age, gender, education, marital status, smoking/drinking history, sleep duration, and life satisfaction scores, based on prior research and established evidence (12). Odds ratios (OR) with 95% confidence intervals (CI) quantified the effects of each ACE on CKDs and DS. Additionally, the marginal effects of ACE count on CKDs, DS, and the co-occurrence of DS and CKDs (DS-CKDs) were also assessed via logistic regression, adjusting for the same covariates.

To explore whether DS mediated the relationship between ACEs and CKD risk, causal mediation analysis was performed, utilizing bootstrap resampling with 1,000 iterations for robustness. Specifically, we decomposed the total effect of ACEs on CKD into direct and indirect effects (mediated through DS). We also performed causal mediation analysis for each of the 12 ACEs as independent variables. To assess whether there were potential exposure-mediator interactions, we examined whether the addition of interactions had a significant impact on the estimated effects compared to models without interactions.

All analyses were conducted in R (Version 4.2.2), with two-tailed *p* values and a significance threshold of *p* < 0.05. Of these, the mediation analysis was performed by the “mediate” function (for causal mediation analysis) from the “Mediation” package (version 4.5.0) in R.

## 3 Results

In this study, a total of 10,247 participants were included, comprising 4,558 (44.5%) males and 5,689 (55.5%) females, with a mean (SD) age of 57.56 (9.00) years. Among these participants, the prevalence of CKD in 2020 was 8.13%. Table 1 provides an overview of participant characteristics. The prevalence of each specific ACE item varied from 0.4% (incarcerated household members) to 32.1% (emotional neglect), as shown in Supplementary Table S1. In total, 80.9% of participants reported at least one ACE among the 12 assessed, while 12.9% experiencing four or more. Compared to those with no ACEs, individuals with four or more ACEs were more commonly male, had lower education levels, and exhibited higher rates of alcohol use, smoking, shorter sleep duration, and reduced life satisfaction.

**Table 1:** Baseline characteristics of participants.

		ACEs, no				
Characteristics		Overall (*n* = 10,247)	0 (*n* = 1,953)	<4 (*n* = 6,970)	≥4 (*n* = 1,324)	*P* value
Gender (%)	Female	5,689 (55.5)	1,172 (60.0)	3,811 (54.7)	706 (53.3)	<0.001^***^
	Male	4,558 (44.5)	781 (40.0)	3,159 (45.3)	618 (46.7)	
Marital status	Married	9,226 (90.0)	1,765 (90.4)	6,270 (90.0)	1,191 (90.0)	0.858
	Unmarried	1,021 (10.0)	188 (9.6)	700 (10.0)	133 (10.0)	
Education level						
Below primary school		4,706 (45.9)	776 (39.7)	3,212 (46.1)	718 (54.2)	<0.001^***^
Primary school		2,218 (21.6)	446 (22.8)	1,492 (21.4)	280 (21.1)	
Middle school		2,190 (21.4)	466 (23.9)	1,499 (21.5)	225 (17.0)	
High school or above		1,133 (11.1)	265 (13.6)	767 (11.0)	101 (7.6)	
Age, years		57.56 (9.00)	57.20 (9.07)	57.63 (8.97)	57.71 (9.02)	0.147
BMI		24.27 (30.96)	24.20 (13.02)	24.13 (22.68)	25.13 (66.81)	0.552
Creatinine		0.75 [0.64, 0.87]	0.75 [0.64, 0.86]	0.75 [0.64, 0.87]	0.76 [0.64, 0.88]	0.089
BUN		15.62 (4.37)	15.25 (4.19)	15.66 (4.37)	15.97 (4.57)	<0.001^***^
Cholesterol		193.52 (38.50)	194.49 (37.71)	193.02 (38.76)	194.69 (38.23)	0.144
LDL		116.43 (34.51)	118.04 (35.31)	116.04 (34.13)	116.11 (35.23)	0.061
Chronic disease						
Hypertension (%)		2,433 (23.7)	440 (22.5)	1,697 (24.3)	296 (22.4)	0.111
Diabetes (%)		533 (5.2)	104 (5.3)	358 (5.1)	71 (5.4)	0.909
Memory disease (%)		101 (1.0)	17 (0.9)	64 (0.9)	20 (1.5)	0.115
Chronic lung disease (%)		831 (8.1)	109 (5.6)	576 (8.3)	146 (11.0)	<0.001^***^
Stroke (%)		187 (1.8)	31 (1.6)	130 (1.9)	26 (2.0)	0.663
Asthma (%)		376 (3.7)	44 (2.3)	258 (3.7)	74 (5.6)	<0.001^***^
Dyslipidemia (%)		949 (9.3)	184 (9.4)	651 (9.3)	114 (8.6)	0.677
Liver disease (%)		290 (2.8)	35 (1.8)	202 (2.9)	53 (4.0)	0.001^**^
Drinker (%)		3,361 (32.8)	566 (29.0)	2,324 (33.3)	471 (35.6)	<0.001^***^
Smoker (%)		3,020 (29.5)	506 (25.9)	2,096 (30.1)	418 (31.6)	0.001^**^
Sleep time, h		7.00 [5.00, 8.00]	7.00 [6.00, 8.00]	6.50 [5.00, 8.00]	6.00 [5.00, 8.00]	<0.001^***^
Life satisfaction		3.06 (0.70)	3.13 (0.68)	3.06 (0.69)	2.93 (0.73)	<0.001^***^

^**^P < 0.01; ^***^P < 0.001. ACEs, adverse childhood experiences; BMI, body mass index; BUN, blood urea nitrogen; LDL, low density lipoprotein.

**Figure 2:**
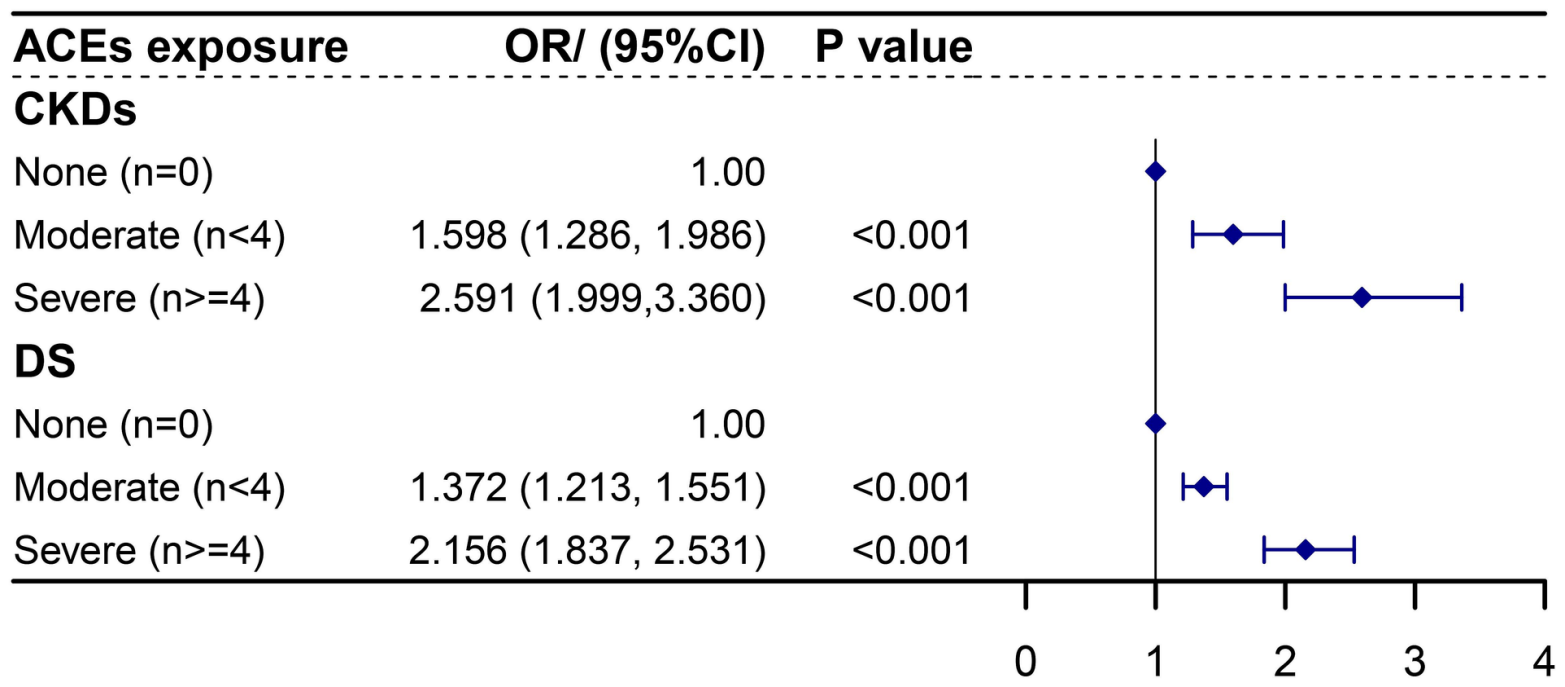
Association of ACEs number with CKDs and DS in adulthood using logistic regression. ACEs, adverse childhood experiences; CKDs, chronic kidney diseases; DS, depression syndrome. Each ACE model was adjusted for age, gender, education level, marital status, smoking and drinking history, sleep duration, and life satisfaction score.

The logistic regression analysis in Figure 2 revealed that individuals with four or more ACEs had a significantly higher risk of CKDs compared to those with no ACE exposure (OR: 2.591, 95% CI: 1.999–3.360). Moderate ACE exposure (fewer than 4 ACEs) also increased CKD risk compared to no exposure (OR: 1.598, 95% CI: 1.286–1.986). The relationships between ACE exposure and DS are further outlined in Figure 2.

**Figure 3:**
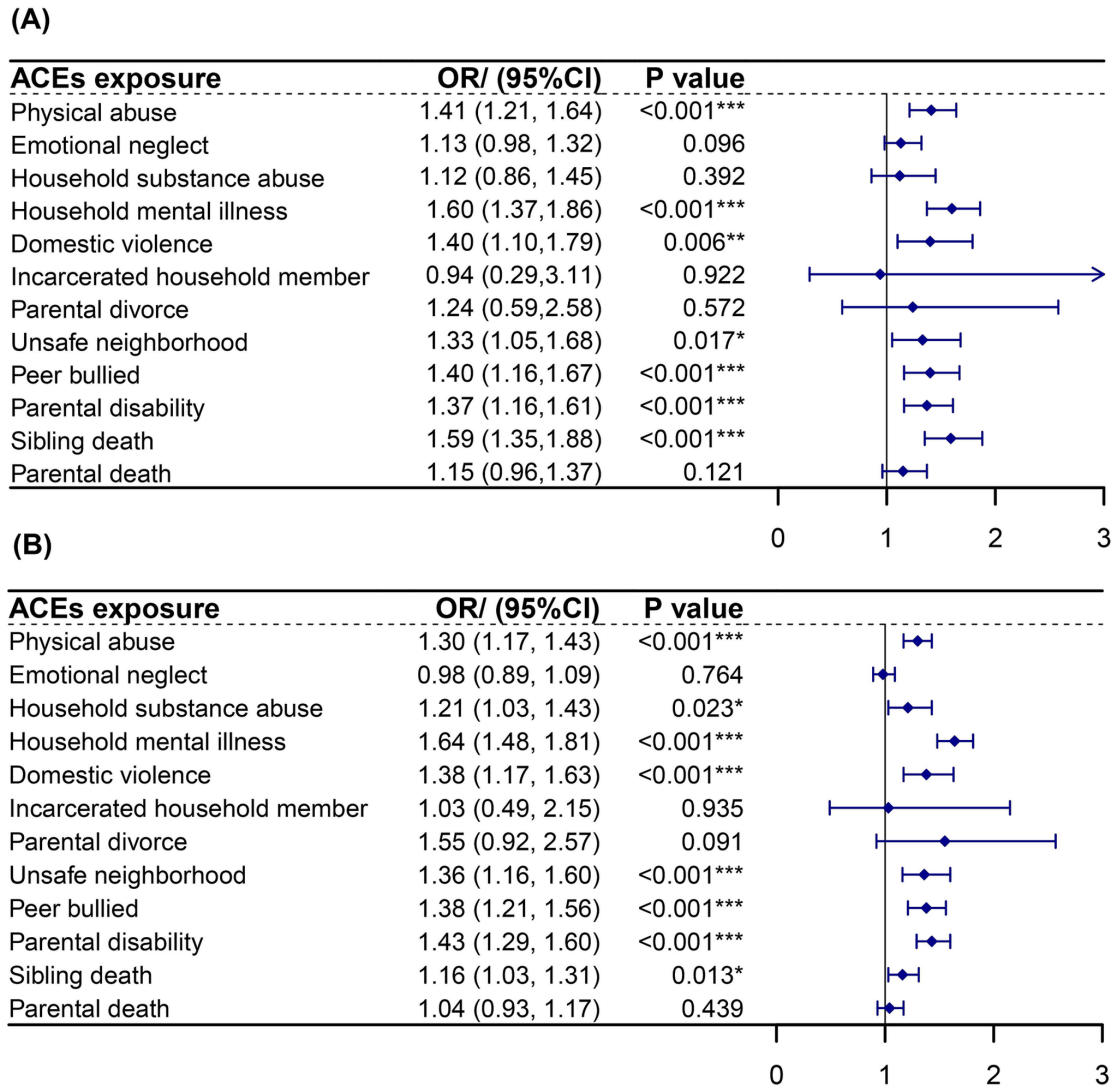
Association of different ACEs exposure with CKDs and DS in adulthood using logistic regression. **(A)** Association of different ACEs exposure with CKDs. **(B)** Association of different ACEs exposure with DS. ^*^p < 0.05; ^**^p < 0.01; ^***^p < 0.001. ACEs, adverse childhood experiences. CKDs, chronic kidney diseases. DS, depression syndrome. Each ACE model was adjusted for age, gender, education level, marital status, smoking and drinking history, sleep duration, and life satisfaction score.

The analysis further illustrates the associations between 12 specific ACE types and the likelihood of experiencing CKDs and DS, after adjusting for confounding factors, as shown in Figure 3. Significant associations with CKDs were observed for physical abuse (OR: 1.41, 95% CI: 1.21–1.64), household mental illness (OR: 1.60, 95% CI: 1.37–1.86), domestic violence (OR: 1.40, 95% CI: 1.10–1.79), unsafe neighborhood (OR: 1.33, 95% CI: 1.05–1.68), peer bullying (OR: 1.40, 95% CI: 1.16–1.67), parental disability (OR: 1.37, 95% CI: 1.16–1.61), and sibling death (OR: 1.59, 95% CI: 1.35–1.88). Similarly, associations with DS were significant for physical abuse (OR: 1.30, 95% CI: 1.17–1.43), household mental illness (OR: 1.21, 95% CI: 1.03–1.43), domestic violence (OR: 1.38, 95% CI: 1.17–1.63), unsafe neighborhood (OR: 1.36, 95% CI: 1.16–1.60), peer bullying (OR: 1.43, 95% CI: 1.29–1.60), parental disability (OR: 1.43, 95% CI: 1.29–1.60), and sibling death (OR: 1.16, 95% CI: 1.03–1.31).

Table 2 displays the marginal associations between ACEs counts and CKDs, DS, and their co-occurrence (DS-CKDs). The marginal analysis demonstrate that moderate ACE exposure (fewer than 4 ACEs) significantly increases the probability of CKDs, DS, and DS-CKDs, with margins of 0.029 (95% CI: 0.017–0.041, *p* < 0.001), 0.053 (95% CI: 0.032–0.075, *p* < 0.001), and 0.023 (95% CI: 0.016–0.029, *p* < 0.001) respectively. Severe ACE exposure (4 or more ACEs) is associated with even higher probabilities: 0.073 (95% CI: 0.052–0.093, *p* < 0.001) for CKDs, 0.142 (95% CI: 0.112–0.171, *p* < 0.001) for DS, and 0.048 (95% CI: 0.035–0.061, *p* < 0.001) for DS-CKDs. These findings indicate a strong, statistically significant correlation between higher ACE counts and increased risks for CKDs, DS, and their comorbid presentation, particularly under conditions of severe exposure.

**Table 2:** Marginal analysis of ACEs with CKDs, DS, and DS-CKDs.

	Margins (95% CI)	SE	Z	*P*
CKDs				
Moderate exposure (*n* < 4)	0.029 (0.017, 0.041)	0.006	4.739	<0.001^***^
Severe exposure (*n* ≥ 4)	0.073 (0.052, 0.093)	0.010	6.940	<0.001^***^
DS				
Moderate exposure (*n* < 4)	0.053 (0.032, 0.075)	0.011	4.962	<0.001^***^
Severe exposure (*n* ≥ 4)	0.142 (0.112, 0.171)	0.016	9.084	<0.001^***^
DS-CKDs				
Moderate exposure (*n* < 4)	0.023 (0.016, 0.029)	0.003	6.666	<0.001^***^
Severe exposure (*n* ≥ 4)	0.048 (0.035, 0.061)	0.007	7.150	<0.001^***^

^***^P < 0.001. ACEs, adverse childhood experiences; CKDs, chronic kidney diseases; DS, depression syndrome; DS-CKDs, Co-occurrence of depression syndrome and chronic kidney diseases.

Given that the mediation analysis results between models with and without interaction terms showed minimal differences (Supplementary Table S2), we conclude that there was no evidence of an exposure-mediator interaction between ACE and DS. This implies that the effect of DS (mediator) on CKD (outcome) remained consistent across varying levels of ACE (exposure), and the direct effect of ACE on CKD also remained stable across different DS levels.

Table 3 presents the mediating effects of DS in the association between ACEs and CKDs. The analysis indicates that DS partially mediates the relationship between ACEs and CKDs under moderate and severe ACE exposure levels. Specifically, under moderate exposure (fewer than 4 ACEs), the indirect effect through DS was 0.003 (95% CI: 0.002, 0.004, *p* < 0.001), while the direct effect was 0.026 (95% CI: 0.014, 0.037, *p* < 0.001), leading to a total effect of 0.029 (95% CI: 0.017, 0.039, *p* < 0.001). For severe exposure (4 or more ACEs), the indirect effect increased to 0.006 (95% CI: 0.002, 0.010, *p* = 0.008), while the direct effect was 0.065 (95% CI: 0.045, 0.085, *p* < 0.001), resulting in a total effect of 0.071 (95% CI: 0.051, 0.091, *p* < 0.001).

**Table 3:** Mediating effects of DS in ACEs and CKDs.

ACEs exposure	Indirect effect	*P*	Direct effect	*P*	Total effect	*P*
	Effect (95% CI)		Effect (95% CI)		Effect (95% CI)	
Moderate exposure (*n* < 4)	0.003 (0.002, 0.004)	<0.001^***^	0.026 (0.014,0.037)	<0.001^***^	0.029 (0.017, 0.039)	<0.001^***^
Severe exposure (*n* ≥ 4)	0.006 (0.002, 0.010)	0.008^**^	0.065 (0.045, 0.085)	<0.001^***^	0.071 (0.051, 0.091)	<0.001^***^
Physical abuse	0.003 (0.002, 0.005)	<0.001^***^	0.023 (0.012, 0.036)	<0.001^***^	0.028 (0.015, 0.040)	<0.001^***^
Emotional neglect	0 (−0.001, 0.001)	0.440	0.009 (−0.003, 0.019)	0.112	0.009 (−0.003, 0.019)	0.124
Household substance abuse	0.002 (0.001, 0.004)	0.044^*^	0.014 (−0.007, 0.035)	0.188	0.016 (−0.005, 0.037)	0.160
Household mental illness	0.007 (0.005, 0.009)	<0.001^***^	0.030 (0.017, 0.043)	<0.001^***^	0.037 (0.024, 0.050)	<0.001^***^
Domestic violence	0.005 (0.003, 0.008)	<0.001^***^	0.023 (0.001, 0.049)	0.040 ^*^	0.029 (0.006, 0.054)	0.016^*^
Incarcerated Household member	0.002 (−0.006, 0.012)	0.776	−0.008 (−0.081, 0.084)	0.732	−0.006 (−0.082, 0.083)	0.756
Parental divorce	0.007 (0.000, 0.016)	0.052	0.011 (−0.053, 0.071)	0.856	0.017 (−0.049, 0.077)	0.716
Unsafe neighborhood	0.005 (0.003, 0.007)	<0.001^***^	0.017 (−0.001, 0.036)	0.072	0.022 (0.003, 0.041)	0.028^*^
Peer bullied	0.005 (0.003, 0.007)	<0.001^***^	0.023 (0.007, 0.039)	0.008 ^**^	0.027 (0.012, 0.044)	<0.001^***^
Parental disability	0.005 (0.004, 0.007)	<0.001^***^	0.021 (0.006, 0.034)	<0.001^***^	0.026 (0.012, 0.040)	<0.001^***^
Sibling death	0.001 (−0.000, 0.003)	0.060	0.037 (0.023, 0.052)	<0.001^***^	0.038 (0.024, 0.053)	<0.001^***^
Parental death	0.000 (−0.001, 0.001)	0.896	0.010 (−0.003, 0.023)	0.12	0.010 (−0.003, 0.023)	0.116

^*^P < 0.05; ^**^P < 0.01; ^***^P < 0.001. ACEs, adverse childhood experiences. Each ACE model was adjusted for age, gender, education level, marital status, smoking and drinking history, sleep duration, and life satisfaction score.

The mediating role of DS was also significant for certain specific types of ACEs. Physical abuse, household mental illness, domestic violence, peer bullying, unsafe neighborhood conditions, parental disability, and sibling death were all associated with significant indirect effects via DS, indicating partial mediation. On the other hand, emotional neglect, parental death, and parental divorce showed no statistically significant indirect or direct effects, suggesting no mediated association with CKDs through DS. Additionally, the results for household substance abuse revealed a marginal indirect effect (0.002, 95% CI: 0.001, 0.004, *p* = 0.044) but did not demonstrate a significant direct or total effect.

## 4 Discussion

The objective of this study was to explore the associations between ACEs, DS, CKDs, particularly focusing on the mediating role of DS. Based on a nationally representative sample of 10,247 middle-aged and older adults in the CHARLS study, we identified that individuals with ACEs exhibited increased risks of both DS and CKD compared to ACE-unexposed counterparts. Mediation analysis further indicated that DS substantially mediated the association between ACEs and CKD development.

In our study, results suggested that ACEs was associated with DS and CKD. In particular, individuals exposed to four or more ACEs face over double the odds of developing CKD compared to those without ACEs. Moderate ACE exposure (fewer than four ACEs) also significantly increased CKD risk. This finding aligns with previous research indicating a link between childhood adversity and CKDs (28, 29). For instance, Zhang et al. (28) reported experiences of adversity in childhood could significantly increase the likelihood of CKD in later life, analyzing a large cohort from the UK Biobank. Another study indicates a significant association between ACEs and decreased renal function, suggesting that ACEs may contribute to kidney disease, while decreased renal function further elevates the risk of all-cause mortality (29). Our study adds to the existing evidence by employing a large sample size and providing data from the Chinese population, thereby enhancing the understanding of ACEs as risk factors for the onset of CKD in later life. We similarly found the association between DS and ACEs: experiencing ACEs significantly increased the risk of DS compared to counterparts without ACE exposure. This is consistent with the findings of a meta-analysis of 43 studies (30). The research of Oshri et al. (31) suggests that ACEs are associated with decreased volume in the right amygdala nuclei, and that decreased volume in the right amygdala nuclei is associated with increased depressive symptoms. This provides a possible explanation for our findings.

Additionally, our results provide insights into the underlying pathways, suggesting that DS may act as a bridge connecting ACEs to CKDs. Prior studies have extensively documented the association between ACEs and DS, and our findings further confirm that ACE exposure in childhood is connected to a greater risk of developing DS and DS-CKDs later in life (18). Marginal analysis highlighted the elevated risk, emphasizing the importance of early interventions to mitigate the effects of ACEs. Interestingly, we found no significant associations between DS and specific ACE types, such as emotional neglect, parental incarceration, parental divorce, or parental death. The lack of significant associations for parental incarceration and parental divorce may be attributed to the relatively low incidence of these experiences within our study population, occurring at rates of only 0.4 and 0.7%, respectively. This limited sample size has likely reduced the statistical power necessary to detect meaningful associations for these specific ACEs.

The mediation analysis indicated that DS partially mediates the effects of both moderate and severe ACE exposures on CKD risk. Specifically, ACE exposure was associated with increased DS levels, which in turn were linked to a heightened risk of CKD. Notably, among individuals with severe ACE exposure, the mediating effect of DS was stronger, with an indirect effect of 0.006. The mediating effect of DS in disease development has been well-documented in prior research. A study by Sonya et al. (32) demonstrated that DS partially mediated the connection between ACEs and coronary heart disease, while health behaviors, including smoking, alcohol dependence, sleep, and physical activity, did not play a significant mediating role.

Several prospective studies have examined additional pathways by which ACEs increase CKD risk, often involving lifestyle-related factors (28, 33–35). ACEs may affect the body’s stress response (33) and increase the body’s susceptibility to chronic inflammation (34), which may lead to kidney injury. This may be a mechanism by which ACEs and CKD are associated. In a prospective cohort study, Li et al. (35) identified BMI, smoking, and hypertension as key mediators in the pathway linking adverse life events from childhood to adulthood with increased CKD risk. In another study, it was noted that childhood adversity is significantly positively associated with the risk of developing CKD, with unhealthy lifestyles potentially serving as partial mediators of this relationship (28). In our study, after adjusting for smoking history, sleep duration, and drinking history, we found that the mediating effect of DS on the ACE-CKD link remained significant. Therefore, DS is important as a pathway to mediating the link between ACE and CKD risk. Public health initiatives should prioritize preventive strategies to mitigate the effects of ACEs, potentially reducing the prevalence of DS and CKD in the older adult population. Integrating mental health support and routine ACE screening within CKD prevention efforts could enhance long-term health outcomes.

Among these ACEs, we also observed significant indirect effects of DS for certain adversities, including physical abuse, household mental illness, domestic violence, peer bullying, and parental disability. These experiences consistently showed partial mediation via DS, suggesting that specific types of adversity exert a particularly strong psychological impact, thereby elevating CKD risk. Furthermore, in these instances of adversity, significant direct effects were also observed, indicating that DS partially, but not fully, mediates the relationship between these ACEs and CKD. This implies potential unidentified factors or pathways underlying the ACE-CKD link, especially regarding sibling death, highlighting the need for additional research.

In contrast, we found that DS fully mediated the association between living in an unsafe neighborhood and CKD. An unsafe neighborhood, characterized by limited access to health resources, increased stressors, and lower social cohesion, has been identified as a CKD risk factor, as demonstrated in prior studies (10, 36). The analysis revealed no evidence of DS serving as a complete mediator in the association between household substance abuse and CKDs.

While our findings support the mediating role of DS in the ACEs-CKD pathway, the observed effect sizes were relatively small in magnitude. This is consistent with prior studies on ACEs and chronic diseases, where individual effect sizes are often modest due to the multifactorial nature of CKD pathogenesis (10). Importantly, even small effects at the individual level may translate to significant population health impacts given the high prevalence of ACEs (37, 38).

Several limitations should be acknowledged. First, CKD was identified solely through self-reported data without medical diagnoses, which may affect accuracy. We addressed this by cross-referencing data from the fourth wave with earlier waves in CHARLS to minimize reporting errors. Second, ACE data were collected retrospectively, raising the possibility of recall bias, particularly for subjective experiences like emotional neglect. Although retrospective measures are prone to bias, research supports their validity when prospective data are unavailable (39). Future studies might enhance measurement precision by employing weighted ACE scores, although previous studies suggest similar associations between unweighted and weighted ACE scores with inflammatory and metabolic health outcomes (40, 41). Unfortunately, this study could not utilize weighted scores due to data constraints. Additionally, we assumed equal risk for all components of ACEs, and the ACE grouping in the analysis was based on cumulative ACE scores. While this approach aligns with established methodologies, using weighted scores might offer greater precision. Third, while categorizing ACEs into 0, 1–3, and ≥4 aligns with prior studies, this approach has limitations. The labels “moderate ACE” (1–3) and “severe ACE” (≥4) may oversimplify the cumulative and interactive nature of ACEs, as different ACE combinations could have distinct biological impacts. Future studies should explore data-driven approaches (e.g., latent class analysis) to refine ACEs classification. In addition, we acknowledge that our cross-sectional assessment of DS limits the ability to distinguish between acute and chronic manifestations of depression. Finally, our study faced potential bias due to a high rate of missing data, which could limit the representativeness of findings.

## 5 Conclusion

In conclusion, this study underscores the significant impact of ACEs on the development of CKDs in middle-aged and older adults, with DS serving as a crucial mediator. These findings highlight the importance of early mental health interventions and ACE-focused prevention strategies to alleviate the burden of CKDs. Policymakers and healthcare professionals should prioritize public health approaches that address childhood adversity as a means of promoting better physical and mental health outcomes across the lifespan.

## Data Availability

Publicly available datasets were analyzed in this study. This data can be found at: https://charls.charlsdata.com/pages/data/111/zh-cn.html.
