# Supplementary material for: The mediating effect of depressive syndrome on the relationship between adverse childhood experiences and chronic kidney diseases among middle-aged and older adults
Source: Front Public Health. 2025 Apr 3;13:1536847. doi: 10.3389/fpubh.2025.1536847 (PMC12003370; doi:10.3389/fpubh.2025.1536847)
Supplement: Supplementary file 1 [file Table_1.docx]

**Supplementary Material**

Supplementary Table S1. Questionnaire Items and Prevalence of Each ACE Indicator.

| Types of ACE | Domain | Questionnaire items | Prevalence |
| --- | --- | --- | --- |
| Conventional ACEs | physical abuse | When you were growing up, did your female/male guardian ever hit you? (often^a^, sometimes^a^, rarely, or never) | 27.90% |
|  | emotional neglect | How much love and affection did your female guardian give you while you were growing up? (often, sometimes, rarely^a^, or never^a^) | 32.10% |
|  |  | How much effort did your female guardian put into watching over you? (a lot, some, a little^a^, or not at all^a^) |  |
|  | household substance abuse | During the years you were growing up, did your female/male guardian ever have alcoholism or drug? (yes^a^ or no) | 7.00% |
|  | household mental illness | Did your female/male guardian have abnormality of mind when you were young? (yes^a^ or no) | 26.10% |
|  |  | During the years you were growing up, had your female/male guardian often showed continued signs of sadness or depression? (during all^a^, most^a^, some, or only a little of the childhood) |  |
|  | Domestic violence | Have your father/mother ever beat up your mother/father? (often^a^, sometimes^a^, not very often, or never) | 7.40% |
|  | Incarcerated household member | During the years you were growing up, have your female/male guardian ever been arrested or sent to prison? (yes^a^ or no) | 0.40% |
|  | Parental separation or divorce | Were your biological parents divorced (including long separation due to emotional problems) before you were 17 years? (yes^a^ or no) | 0.70% |
| Expanded ACEs | Unsafe neighborhood | Was it safe being out alone at night in the neighborhood where you lived as a child? (very safe, somewhat safe, not very safe^a^, or not safe at all^a^) | 8.30% |
|  | Peer bullied | When you were a child, how often were you picked on or bullied by kids in your neighborhood? (often^a^, sometimes^a^, not very often, or never) | 14.80% |
| New ACEs | Parental disability | Did your female/male guardian have a long time being sick on bed when you were young? (yes^a^ or no) | 20.70% |
|  |  | Did your female/male guardian have a serious deformity when you were young? (yes^a^ or no) |  |
|  | Sibling death | Any of the siblings was dead before participant was 17 years. (yes^a^ or no) | 17.00% |
|  | Parental death | Either of the parents was dead before participant was 17 years. (yes^a^ or no) | 18.50% |

Abbreviation: ACEs: adverse childhood experiences.

^a^ Answers indicate thresholds for ACEs.

^b^ Calculated based on dates of birth and their parental death.

^c^ Calculated based on dates of birth and their sibling's death.

Supplementary Table S2. Mediation analysis results comparing models with versus without exposure-mediator interaction for the ACEs-CKD relationship, using DS as a mediator.

| **ACEs exposure** | **Indirect effect**  **Effect (95% CI)** | **P** | **Direct effect**  **Effect (95% CI)** | **P** | **Total effect**  **Effect (95% CI)** | **P** |
| --- | --- | --- | --- | --- | --- | --- |
| ***Model without exposure-mediator interaction*** | | | | | | |
| Moderate exposure | 0.003 (0.002, 0.004) | <0.001*** | 0.026 (0.014,0.037) | <0.001*** | 0.029 (0.017, 0.039) | <0.001*** |
| Severe exposure | 0.006 (0.002, 0.010) | 0.008** | 0.065 (0.045, 0.085) | <0.001*** | 0.071 (0.051, 0.091) | <0.001*** |
| ***Model with the exposure-mediator interaction*** | | | | | | |
| Moderate exposure | 0.002 (0.001, 0.004) | <0.001*** | 0.027 (0.015,0.038) | <0.001*** | 0.029 (0.018, 0.040) | <0.001*** |
| Severe exposure | 0.006 (0.002, 0.009) | <0.008*** | 0.065 (0.046, 0.085) | <0.001*** | 0.071 (0.051, 0.091) | <0.001*** |

**: P < 0.01; ***: P < 0.001.

ACEs: adverse childhood experiences.

Each ACE model was adjusted for age, gender, education level, marital status, smoking and drinking history, sleep duration, and life satisfaction score.
